# Molecular detection of *Mycobacterium tuberculosis* from buccal swabs among adult in Peru

**DOI:** 10.1038/s41598-020-79297-9

**Published:** 2020-12-17

**Authors:** Annelies W. Mesman, Roger I. Calderon, Nira R. Pollock, Martín Soto, Milagros Mendoza, Julia Coit, Zibiao Zhang, Juan Aliaga, Leonid Lecca, Rebecca C. Holmberg, Molly F. Franke

**Affiliations:** 1 Department of Global Health and Social Medicine, Harvard Medical School, Boston, USA; 2 Socios En Salud Sucursal (Partners In Health), Lima, Peru; 3 Faculdade de Medicina, Programa Acadêmico de Tuberculose, Universidade Federal do Rio de Janeiro, Rio de Janeiro, 21941-590 Brazil; 4 Department of Laboratory Medicine, Boston Children’s Hospital, Boston, USA; 5 Akonni Biosystems Inc., Frederick, USA

**Keywords:** Clinical microbiology, Diagnosis

## Abstract

Tuberculosis (TB) diagnosis relies on a sputum sample, which cannot be easily obtained from all symptomatic patients. *Mycobacterium tuberculosis* DNA can be detected from oral swabs, a noninvasive, safe alternative sample type; however, reported sensitivities have been variable and likely depend on sample collection, processing procedures and host characteristics. We analyzed three buccal swab samples from 123 adults with culture-confirmed TB in Lima, Peru. We compared the sensitivity and specificity of two sample collection devices (OmniSwab and EasiCollect FTA cards) and examined factors associated with detection. DNA was extracted with a commercially available kit and detected via real-time PCR IS6110 amplification. Overall sensitivity for buccal samples was 51% (95% Confidence Interval [CI] 42–60%). Specificity from a single sample among healthy controls was 96.7% (95% CI 83–99.9%). Positive sputum smear and cavitary disease, correlates of disease burden, were associated with detection via buccal swab. Although we observed higher sensitivities with the Omniswab samples, this appeared to be due primarily to differences in patient characteristics (e.g., cavitary disease). Overall, our findings support the potential for a buccal sample-based TB assay. Future work should focus on assay optimization and streamlining the assay workflow.

## Introduction

Tuberculosis (TB) disease remains a global health burden, with an estimated 10 million cases in 2019^1^. Prompt diagnosis and treatment initiation are critical to TB care and control, but are often limited for patients who are unable to produce a sputum sample for microbiological confirmation of *Mycobacterium tuberculosis* (*Mtb*) (e.g., children and some individuals living with HIV)^2,3^.

Because obtaining a sputum sample is not possible in all patients, studies have sought to identify alternative sample types for molecular detection of TB disease, including oral swabs^4–7^. This specimen is advantageous because collection is non-invasive and reduces transmission risk to health care workers as it produces no aerosols.

Studies of oral swab samples among adults and children in South Africa have yielded high sensitivity and specificity^4,8,9^. Studies in Peru by our team have found lower test sensitivities^10,11^, potentially due to different collection and detection methods, collection of a single sample (versus two or three), and differences in patient populations (varying disease burdens and co-morbidities)^8,10,11^. Thus, outstanding questions about optimal sampling and detection strategies remain and should be researched among patients in whom TB can be reliably diagnosed before translation to populations in which microbiologic confirmation is more difficult. Here, we report detection of *Mtb* from buccal samples collected from adults with culture-confirmed TB disease in Lima, Peru. We compare sensitivity and specificity of two collection devices: the OmniSwab, a swab that is stored and transferred in buffer, and the EasiCollect, a foam swab that transfers sample onto an FTA card for transport and storage. We also examined patient and sample collection factors associated with detection.

## Materials and methods

### Ethical considerations

This study was approved by the Ethics Committee of Peru’s National Institute of Health and the Office of Human Research Administration at Harvard Medical School. Study participants provided written informed consent. All methods were carried out in accordance with approved guidelines and relevant regulations.

### Study population

These data were collected as part of a larger diagnostic project and collection of oral swab samples began midway through the study. We recruited adults, at least 18 years of age, that were diagnosed with culture-confirmed TB disease through the National Tuberculosis Program in Ministry of Health centres in Lima, Peru.

All patients provided a pre-treatment sputum sample for research purposes for smear analysis, culture, and drug-sensitivity testing. Additionally, study staff collected data from the patient’s medical chart (history of prior TB treatment, diabetes, and chest radiography results) and conducted voluntary HIV testing. We aimed to collect three buccal swab samples per patient. The first was collected prior to treatment initiation and the other two samples were collected leveraging protocolized study visits occurring during the first week of treatment (days 4 and 7). Because the larger diagnostic study did not enroll symptomatic adults in whom TB had been ruled out (i.e., the ideal control group), we collected single buccal swab samples from healthy controls recruited from the same health centres, which they visited for reasons unrelated to lung health.

### Sample collection and testing procedures

We collected buccal swab samples using one of two collection devices: (1) the OmniSwab (Whatman, catalogue #WB100035) and (2) the EasiCollect FTA card device (Whatman catalogue #WHAWB120210). Samples were collected by trained staff, who gently brushed the inside of each cheek of the participants for 10 s with the OmniSwab or the EasiCollect sponge attached to the FTA card. The OmniSwab has a breakpoint and the head was ejected into 500 µl buffer containing 50 mM Tris pH 8.0, 50 mM EDTA, 50 mM sucrose, 100 mM NaCl, and 1% SDS, and transported to the laboratory at 4^○^C. There, the OmniSwab-collected samples were vortexed, and the swabs heads removed, followed by storage at – 80 °C until further processing. Sponges from EasiCollect devices were pressed onto the FTA cards, which were placed into small plastic bags according to manufacturer’s guidelines, transported at room temperature, and stored at − 20 °C until processing. For each participant, we aimed to collect all three oral samples with the same type of collection device. When possible, study staff alternated the collection device type for each new enrollee; however, swab supply shortages and importation delays routinely interfered with this assignment strategy.

Laboratory procedures including smear microscopy, culture, and nucleic acid extraction, were carried out in the Socios En Salud Sucursal Peru Biosafety Level 3 Laboratory in Lima, Peru. Sputum was decontaminated using 2% NaOH/ 0.25% *N*-acetyl-L-cysteine (NALC) prior to smear microscopy (Ziehl–Neelsen staining) and culture (BACTEC MGIT 960, BD Franklin Lakes, USA).

The FTA card was cut into pieces and placed in a tube with 300 µl 0.1 N NaOH, 0.3 mM EDTA, pH 13.0 solution for five minutes at room temperature. Next, 300 µl of 0.1 M Tris–HCl, pH 7.0 was added to neutralize, followed by a heating step at 90 °C for 20 min to release DNA from the card. The sample was then processed through a Mini spin column (QIAamp DNA Mini Kit (Qiagen, MD, USA)). OmniSwab samples in lysis buffer were treated according to the protocol described by Wood et al.^4^. This procedure included incubation of the full 500 µl sample volume at 95 °C for 10 min before proceeding with extraction using the QIAamp DNA Mini Kit (Qiagen, MD, USA). For both methods, DNA was eluted in 50 µl of elution buffer pre-warmed to 42ºC. Real-time PCR analyses (qPCR; Roche 480 II Lightcycler System; Basel, Switzerland) amplifying the IS6110 target^12^ was performed in duplicate using 5 µl of each extract. The only exception to the protocol by Wood et al.^4^ was the lack of a further ethanol precipitation for negative samples. With every batch of samples, a negative and positive extraction control was included. We used a 1:1000 dilution of *Mtb* H37Ra cells (0.5 McFarland standard) for the positive control. The negative control was lysis buffer for OmniSwab and DNAse free water for EasiCollect samples.

We defined *Mtb* detection in a sample if both qPCR replicates had a crossing point (Cp) value of < 40 and an increase in fluorescence greater than 10 units within 40 cycles. Discordant replicates were repeated in duplicate on qPCR. Technicians were blinded to culture results and clinical information.

### Data analysis

A patient was classified as positive for TB by oral swab if qPCR detection was positive for both replicates for at least one of the patient’s buccal samples tested. Sensitivity and specificity were calculated among cases and controls, respectively, along with 95% confidence intervals (CI). We conducted sensitivity analyses per sample and per patient and adjusted per sample confidence intervals for clustering by patient. We estimated sensitivity overall and stratified by collection device (i.e. OmniSwab or EasiCollect), time of collection (pre or post treatment), and smear result.

To examine associations between *Mtb* detection in buccal swab samples and factors related to sample collection (i.e., collection device, pre- versus post-TB treatment initiation), disease burden (i.e., smear positivity, cavitary disease) and comorbidity (i.e., diabetes mellitus), we conducted binomial generalized estimating equation (GEE) regression analyses. Multivariable analyses adjusted for all of the above factors. We adjusted analyses for clustering by patient using an unstructured correlation structure. Chest radiography results, and hence cavitary disease data, were missing for six patients. Therefore, we added a missing data indicator to the multivariate model for this variable. Statistical analyses were conducted in SAS (Version 9.4. SAS Institute Inc., Cary, NC, USA). We applied the recommendations by Cole et al. for numerical precision for p-values, percentages, risk ratios and confidence intervals^13^.

## Results

### Study population

**Figure 1:**
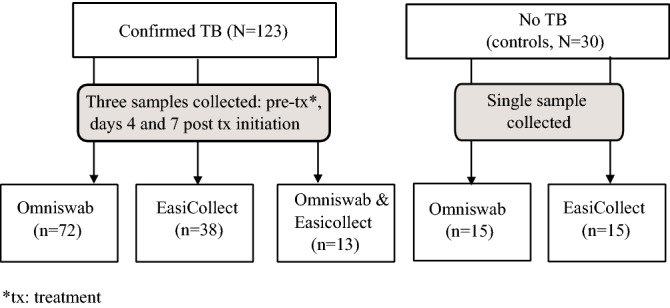
Flowchart of participant enrollment and oral swab sample collection method.

We analysed samples from 123 patients, from whom we had three (n = 106), two (n = 15) or one (n = 2) buccal samples (Fig. 1). Patient characteristics are presented in Table 1. The median age among cases was 32 years; 9.8% self-reported diabetes and 3.3% were living with HIV. A majority of patients had a positive sputum smear (64%) and/or a chest radiography indicating cavitary disease (76%). For most patients (n = 110; 89%), the collection device was the same for all samples (72 patients with OmniSwab and 38 patients with EasiCollect; Fig. 1). Thirteen patients had their samples collected with a combination of OmniSwab and EasiCollect (Table 1; Fig. 1; Supplementary Table 1).

**Table 1:** Demographic and clinical characteristics of patients with TB, stratified by buccal sample collection method.

		All (N = 123)	OmniSwab (N = 72)	EasiCollect (N = 38)	Combination OmniSwab/EasiCollect (N = 13)
Age	Median, Q1–Q3	32 (23–44)	32 (23–43)	33 (23–45)	25 (25–43)
Sex	Female	41 (33)	25 (35)	27 (71)	5 (38)
	Male	82 (67)	47 (65)	11 (29)	8 (62)
Diabetes	Yes	12 (9.8)	8 (11)	3 (7.9)	1 (7.7)
	No	111 (90)	64 (89)	35 (92.1)	12 (92.3)
HIV	Yes	4 (3.3)	3 (4.2)	1 (2.6)	0 (0)
	No	119 (96.7)	69 (95.8)	37 (97.4)	13 (100)
Smear-status	Positive	79 (64)	45 (62)	25 (66)	9 (69)
	Negative	44 (36)	27 (38)	13 (34)	4 (31)
Cavitary disease*	Yes	89 (76)	52 (78)	25 (68)	12 (92.3)
	No	28 (24)	15 (22)	12 (32)	1 (7.7)
MDR-TB**	Yes	11 (10)	7 (12)	2 (5.3)	2 (18)
	No	98 (90)	53 (88)	36 (94.7)	9 (82)

Results are expressed as n (%), unless otherwise noted.

*6 missing.

**14 missing.

### Sample-level analyses of sensitivity

We detected *Mtb* in 26% of the buccal samples from patients with TB (95% Confidence Interval [CI] 21–32%) (Table 2). Sensitivity was 30% among samples collected with OmniSwab (95% CI 23–37%) and 20% among samples collected with EasiCollect (95% CI 11–28%). Among patients with smear-positive TB, per sample sensitivity was 34% (95% CI 26–42%) as compared to 14% in patients with smear-negative TB (95% CI 8.2–19%).

**Table 2:** Detection of *Mycobacterium tuberculosis* per sample (upper panel) and per patient (lower panel).

By sample	All samples		OmniSwab		EasiCollect			
	Positive result (n/N)	Sensitivity (95% CI^a^)	Positive result (n/N)	Sensitivity (95% CI)	Positive result (n/N)	Sensitivity (95% CI)		
All samples	92/350	26 (21–32)	67/223	30 (23–37)	25/127	20 (11–28)		
All Sm^b^ + TB	74/219	34 (26–42)	55/137	40 (30–50)	19/82	23 (11–35)		
All Sm − TB	18/131	14 (8.2–19)	12/86	14 (6.6–21)	6/45	13 (4.0–23)		
Pre-tx^c^ samples	23/107	22 (14–29)	18/63	29 (17–40)	5/44	11 (1.6–21)		
Post-tx samples	69/243	28 (22–35)	49/160	31 (22–39)	20/83	24 (13–35)		

By patient	All patients		OmniSwab		EasiCollect		Combination OmniSwab/EasiCollect	
	Positive result (n/N)	Sensitivity (95% CI^a^)	Positive result (n/N)	Sensitivity (95% CI)	Positive result (n/N)	Sensitivity (95% CI)	Positive result (n/N)	Sensitivity (95% CI)
All patients	63/123	51 (42–60)	38/72	53 (41–64)	15/38	39 (24–55)	10/13	77 (46–95)
All Sm^b^ + TB	46/79	58 (47–69)	29/45	64 (50–78)	10/25	40 (21–59)	7/9	78 (40–97)
All Sm − TB	17/44	39 (24–53)	9/27	33 (16–51)	5/13	39 (14–68)	3/4	75 (19–99.4)

^a^*CI* confidence interval.

^b^*SM* smear-microscopy.

^c^*tx* treatment.

### Patient-level analyses of sensitivity

*Mtb* was detected in at least one buccal sample in 51% of patients (95% CI 42–60%; Table 2). Among the 106 patients from whom we had collected 3 samples, sensitivity was 47% (95% CI 37–57%). Sensitivity was 53% among 72 patients who had all samples collected with OmniSwab (95% CI 41–64%) and 39% among 38 patients who had all samples collected with EasiCollect (95% CI 24–55%). We found a sensitivity of 58% among patients with smear-positive TB (95% CI 47–69%), this was highest, 64% (95% CI 50–78%; Table 2), among patients who had all samples collected with OmniSwab. Among patients with smear-negative TB sensitivity was 39% (95% CI 24–53%; Table 2). While sensitivity (77%; 95% CI 46–95%) was higher among the 13 patients with samples collected with both devices, confidence intervals were wide (Table 2; data shown in Supplementary Table 1). Testing multiple samples increased detection for both collection methods (Table 3). After a single sample, test sensitivity was 31% among patients in the OmniSwab and 21% in the EasiCollect group, which increased to 53% and 39% sensitivity after three samples respectively (Table 3).

**Table 3:** *Mycobacterium tuberculosis* detection yield per time point and cumulatively for the 110 patients who had samples collected with either the OmniSwab or EasiCollect device.

	Pre-treatment sample n/N (%)	Post-treatment sample 1* n/N (%)	Post-treatment sample 2** n/N (%)
**Per time point**			
OmniSwab	22/72 (31)	21/71 (30)	16/62 (26)
EasiCollect	8/38 (21)	8/37 (22)	4/32 (13)
**Cumulative**			
OmniSwab	22/72 (31)	32/72 (44)	38/72 (53)
EasiCollect	8/38 (21)	12/38 (32)	15/38 (39)

*Post-treatment sample 1 was typically collected 4 days following treatment initiation.

**Post-treatment sample 1 was typically collected 7 days following treatment initiation.

### Specificity

Based on a single buccal sample from 30 controls we observed an overall specificity of 96.7% (95% CI 83–99.9%). Specificity per collection device was 93.3% (95% CI 68–99.8%; 1/15 samples tested positive) and 100% (95% CI 78–100%; 0/15 samples tested positive) for EasiCollect and OmniSwab, respectively.

### Factors associated with Mtb detection among patients with TB

Sample collection using the OmniSwab was positively associated with *Mtb* detection relative to collection with the EasiCollect device in univariate analyses (RR: 1.65; 95% CI 1.0–2.7, p = 0.05), though this was attenuated in multivariate analyses (RR: 1.38 95% CI 0.9–2.1, p = 0.16). There was no association between time of collection and *Mtb* detection. Correlates of disease burden (i.e., sputum smear positivity, cavitary disease) were strongly associated with detection of *Mtb* in buccal samples (Table 4). In univariate analyses, buccal swab samples collected from patients with a positive sputum smear and/or cavitary disease were more likely test positive for *Mtb*, respectively (risk ratio [RR] for positive sputum smear: 2.51 (95% CI 1.6–3.9, p < 0.0001); RR for cavitary disease: 4.5 (95% CI 2.1–9.5, p < 0.0001) and this remained true in multivariable analysis (RR: for positive sputum smear 2.05 (95% CI 1.3–3.2, p = 0.001); RR for cavitary disease was 3.28 (95% CI 1.6–6.6, p = 0.0008)). Having diabetes was also positively associated with detection (univariate RR: 1.67; 95% CI 1.0–2.7, p = 0.04; multivariate RR: 1.58; 95% CI 1.1–2.3, p = 0.02). We did not include HIV in our analysis due to the low number of patients living with HIV in our study cohort (N = 4).

**Table 4:** Factors associated with *Mycobacterium tuberculosis* detection in 350 buccal samples.

		Univariate RR^a^ (95% CI^b^)	p-value	multivariate RR (95% CI)	p-value
Swab collection	OmniSwab (vs EasiCollect)	1.65 (1.0–2.7)	0.05	1.38 (0.9–2.1)	0.16
	Post-tx^c^ sample collection (vs pre-tx)	1.29 (0.9–1.9)	0.17	1.32 (0.9–1.9)	0.17
Disease burden	Positive smear-status	2.51 (1.6–3.9)	< 0.0001	2.05 (1.3–3.2)	0.001
	Cavitary disease^d^	4.5 (2.1–9.5)	< 0.0001	3.28 (1.6–6.6)	0.0008
Comorbidity	Diabetes	1.67 (1.0–2.7)	0.04	1.58 (1.1–2.3)	0.02

^a^*RR* risk ratio.

^b^*CI* confidence interval.

^c^*tx* treatment.

^d^The univariate model for cavitary disease and multivariate model includes a missing indicator for the six people (18 samples) for whom X-ray was missing.

## Discussion

These methods for buccal swab collection, processing, and analysis yielded a sensitivity of 51% in 123 adult TB patients. Among patients with smear-positive TB whose samples were collected with the OmniSwab, observed sensitivity reached 64% [95% CI 50–78%]. These sensitivities are lower than the sensitivities of 83 and 90% reported in studies from South Africa^4,8^, which used a similar molecular assay and collected two swabs from each adult with confirmed TB. Differing bacillary burdens between the two population is one potential explanation. We included culture-confirmed patients whereas the South African studies included Xpert-confirmed TB cases. Because Xpert is less sensitive than culture^14^, use of Xpert as the reference standard could have selected for a study population with a higher bacillary load. One of the two South African studies included 10 patients with Xpert-negative yet culture-positive sputum results and oral swab analysis detected *Mtb* in only four of 10 (40%) patients in this group^8^ (as compared to 128 of 138 (93%) of patients who were Xpert-positive). While that study did not report data on sputum smear results or chest X-ray findings^8^, this explanation for varying sensitivities is consistent with our observation that detection of *Mtb* from buccal swabs is associated with extent of disease, as measured by smear positivity and cavitary disease.

Alternatively, the higher sensitivity in the other studies could be related to differences between procedures, either in sample collection or the molecular assay. For example, the flocked swab identified as optimal in the study by Luabeya et al. is different from the plastic, non-bristled swab used in our study^8^. And, the EasiCollect depends on efficient transfer of the sample from the sponge to the FTA card, which can be dependent on the pressure applied by the user. Swabbing the tongue instead of the cheek, use of ethanol precipitated nucleic extracts or increasing the number of amplification cycles may also improve detection^4,8^.

Type 2 diabetes mellitus is a risk factor for TB disease and unfavorable treatment outcomes^15,16^. Our study found a positive association between swab detection and diabetes, while Wood et al. reported a negative association between the two^4^. Further study to evaluate whether and how diabetes influences *Mtb* detection from oral swabs may be relevant, since diabetes affects oral mucosa and salivary gland function, which potentially affects *Mtb* presence and detection in oral swabs^17^.

Our findings contribute to the small body of evidence around oral swab testing in several ways. First, various factors, including disease burden and diabetes, affected the sensitivity of oral swab analyses—this has important implications for sample utility and reporting of findings. Sensitivity was lower in adults with smear-negative disease. Given that children are more likely than adults to have smear-negative TB, oral swab sensitivity would need to be optimized in order to be reliable in the group most in need of a diagnostic. Future oral swab studies may wish to report sensitivity stratified by factors that predict detection, in order to facilitate comparisons across studies. We also compared two swab types and found weak evidence that OmniSwabs had a higher test sensitivity compared to samples collected with the EasiCollect device. Flores et al. reported a similar finding in a paediatric study^11^. If the benefits of an FTA card-based collection method, which doesn’t require cold-chain transport or buffer preparation, are considered critical for implementation in low-resource settings, this collection method may require optimization. Overall, our findings suggest that sampling strategies and procedures, as well as patient characteristics should be considered in the design, analysis, and interpretation of future oral swabs studies.

Our study has several limitations. First, we only included a small asymptomatic control group from whom we collected a single swab. Therefore, the specificity cannot be estimated precisely nor in the group most relevant for implementation (i.e., patients with respiratory symptoms who are determined not to have TB). Because specificity of *Mtb* detection from oral swabs has been shown to be high consistently^4,9,11^, we focused on sample sensitivity and the factors that drive it. Second, patients were assigned to the OmniSwab or EasiCollect groups based on availability of the materials rather than randomization, raising the possibility of confounding by patient characteristics. Ideally, participants would be randomized or samples would be collected by both methods from each participant. We aimed to account for this potential bias by adjusting for indicators of disease severity in analyses of swab type.

Overall, the buccal sample procedures we present in this paper are promising but require further optimization to be useful. We advocate for future research to develop an oral sample-based TB assay that relies on less labour-intensive nucleic acid extraction and detection methods^18^ and has high sensitivity in patients with smear-negative TB disease, which is especially common in patient groups not able to spontaneously produce a sputum sample.

## Supplementary Information

Supplementary Table 1.
