# Supplementary material for: Molecular detection of Mycobacterium tuberculosis from buccal swabs among adult in Peru
Source: Sci Rep. 2020 Dec 17;10:22231. doi: 10.1038/s41598-020-79297-9 (PMC7746708; doi:10.1038/s41598-020-79297-9)
Supplement: Supplementary file 1 — Supplementary Table 1. [file 41598_2020_79297_MOESM1_ESM.pdf]

Supplementary Information

Article title:

Molecular detection of *Mycobacterium tuberculosis* from buccal swabs among adult in Peru

Authors:

Annelies W Mesman[1*], Roger I Calderon[2,3], Nira R Pollock[4], Martín Soto[2], Milagros Mendoza[2], Julia Coit[1], Zibiao Zhang[1], Juan Aliaga[2], Leonid Lecca[2], Rebecca C Holmberg[5], Molly F Franke[1]

[1] Department of Global Health and Social Medicine, Harvard Medical School, Boston, USA

[2] Socios En Salud Sucursal (Partners In Health), Lima, Peru

[3] Programa Acadêmico de Tuberculose. Faculdade de Medicina. Universidade Federal do Rio de Janeiro, Rio de Janeiro 21941-590, Brazil

[4] Department of Laboratory Medicine, Boston Children's Hospital, Boston, USA,

[5] Akonni Biosystems Inc, Frederick, USA.

*Corresponding author: anneliesmesman@gmail.com

Supplementary Table S1. Collection methods and results for the 13 patients whose buccal swab sample were collected with a combination of the OmniSwab and EasiCollect device.

		Sample 1		Sample 2		Sample 3	
**Patient**	**Smear result**	**Method**	**Result**	**Method**	**Result**	**Method**	**Result**
1	-	EasiCollect	Negative	OmniSwab	Negative	EasiCollect	Negative
2	+++	OmniSwab	Positive	EasiCollect	Positive		
3	-	EasiCollect	Positive	EasiCollect	Negative	OmniSwab	Negative
4	+	OmniSwab	Negative	EasiCollect	Negative	EasiCollect	Negative
5	+	EasiCollect	Positive	OmniSwab	Positive	EasiCollect	Negative
6	+	OmniSwab	Negative	EasiCollect	Negative	EasiCollect	Negative
7	+	EasiCollect	Positive	EasiCollect	Positive	OmniSwab	Negative
8	1-9	EasiCollect	Negative	OmniSwab	Positive	EasiCollect	Negative
9	+	OmniSwab	Positive	EasiCollect	Negative	OmniSwab	Negative
10	-	EasiCollect	Negative	OmniSwab	Positive	OmniSwab	Negative
11	-	EasiCollect	Negative	OmniSwab	Positive	OmniSwab	Negative
12	+	EasiCollect	Negative	OmniSwab	Positive	OmniSwab	Negative
13	+++	EasiCollect	Negative	OmniSwab	Positive	OmniSwab	Negative
